# Etiology-Dependent Microbiome Differences in Hepatocellular Carcinoma Development

**DOI:** 10.3390/ijms252413510

**Published:** 2024-12-17

**Authors:** Nevena Todorovic, Serena Martinelli, Giulia Nannini, Ralf Weiskirchen, Amedeo Amedei

**Affiliations:** 1 Department of Experimental and Clinical Medicine, University of Florence, 50134 Florence, Italy; nevena.todorovic@unifi.it (N.T.); serena.martinelli@unifi.it (S.M.); giulia.nannini@unifi.it (G.N.); 2 Clinic for Infectious and Tropical Diseases, University Clinical Centre of Serbia, 11000 Belgrade, Serbia; 3 Institute of Molecular Pathobiochemistry, Experimental Gene Therapy and Clinical Chemistry (IFMPEGKC), RWTH University Hospital Aachen, D-52074 Aachen, Germany; 4 Network of Immunity in Infection, Malignancy and Autoimmunity (NIIMA), Universal Scientific Education and Research Network (USERN), 50139 Florence, Italy

**Keywords:** gut microbiota, oncobiome, fibrosis, steatosis, cirrhosis, hepatocellular carcinoma

## Abstract

Chronic liver disease is characterised by persistent inflammation, tissue damage, and regeneration, which leads to steatosis, fibrosis, and, lastly, cirrhosis and hepatocellular carcinoma (HCC). HCC, the most prevalent form of primary liver cancer, is one of the leading causes of cancer-related mortality worldwide. The gut microbiota plays a fundamental role in human physiology, and disturbances in its critical balance are widely recognised as contributors to various pathological conditions, including chronic liver diseases, both infectious and non-infectious in nature. Growing interest in microbiota research has recently shifted the focus towards the study of intratumoural microbiota, referred to as the “oncobiome”, which can significantly impact the development and progression of HCC. In this review, we discuss existing research and provide an overview of the microbiota influence on viral hepatitis, particularly in shaping the progression of liver disease caused by the hepatitis B and hepatitis C viruses. We also explore microbial dysbiosis and its contribution to the silent and dangerous progression of non-alcoholic fatty liver disease. Additionally, we address the impact of alcohol on the liver and its interaction with the microbiota, tracing the pathway from inflammation to cirrhosis and cancer. The review emphasises the most common etiologies of hepatocellular carcinoma.

## 1. Introduction: The Fragile Liver and the Hidden Ecosystem

Chronic liver disease (CLD) refers to the gradual decline in liver function over a period exceeding six months. This process is characterised by ongoing inflammation, tissue damage, and regeneration, which leads to steatosis, fibrosis, and, lastly, cirrhosis and hepatocellular carcinoma. These stages do not always follow a linear progression, and several may occur simultaneously in the same individual. Diverse causes are linked to CLD, including metabolic disorders, infections, prolonged alcohol use, chronic exposure to toxins (such as pesticides, aflatoxins, and microcystins), autoimmune conditions, and genetic disorders. Its progression and severity are influenced by genetic factors even among subjects with the same underlying conditions, but an additional factor is increased age, while women generally experience slower advancement, except in cases of alcoholic liver disease (ALD) [1,2,3].

Liver fibrosis, as a pathological response to prolonged liver injuries, is defined by the overproduction and accumulation of extracellular matrix (ECM) and abnormal connective tissue growth. Chronic damage leads to the destruction of hepatocyte membranes, resulting in necrosis, apoptosis, and the scarring of liver tissue. Upon injury, hepatocytes release damage-associated molecular patterns, which activate quiescent hepatic stellate cells (HSCs), shifting them into a fibrogenic state, leading to exaggerated ECM production, primarily composed of type I and type III collagen and fibronectin. As a consequence, the equilibrium between matrix metalloproteinases (MMPs), responsible for ECM degradation, and tissue MMPs inhibitors, becomes disrupted. This causes ECM accumulation and the development of scar tissue, compromising the liver’s structural integrity and so impairing its normal functions. The mechanisms driving HSC survival and activation, while promoting inflammation and worsening tissue damage, include: (1) autocrine activation of HSCs through cytokines such as transforming growth factor beta-1 (TGF-β1), platelet-derived growth factor (PDGF), and cellular communication network factors, (2) HSCs secrete chemokines that perpetuate the inflammatory response, (3) activated Kupffer cells and other immune cells release PDGF, TGF-β1, tumor necrosis factor-alpha (TNF-α), and interleukin-1 beta (IL-1β), (4) Kupffer cells secrete chemokines, like CCL2 and CCL5, which recruit monocytes to the inflamed liver tissue, which in turn, produce apoptosis-signal-regulating kinase 1, pan-caspase, and galectin-3 among other mediators, and finally (5) recruited monocytes differentiate into macrophages, which then release IL-1 and IL-6. Chronic viral infections typically result in portal and periportal fibrosis, while ALD and non-alcoholic fatty liver disease (NAFLD) often start with centrilobular fibrosis. A study involving 4852 patients highlighted varying rates of fibrosis progression, with the fastest rates seen in human immunodeficiency virus (HIV)-hepatitis C virus (HCV) co-infected patients and the slowest in primary biliary cirrhosis. Cirrhosis represents the advanced CLD stage, characterised by disrupted liver architecture, nodule formation, vascular reorganisation, and ECM deposition [3,4,5,6,7,8,9].

HCC, the most common form of primary liver cancer, is a leading cause of cancer-related death worldwide [10,11]. It poses a significant global health challenge due to its widespread risk factors and poor prognosis. The pathophysiology of HCC is complex and progresses through multiple stages, involving gene mutations, dysregulation of signalling pathways, epigenetic changes, hepatitis B virus (HBV)-induced carcinogenesis, chronic inflammation, tumour microenvironment (TME) perturbations, and oxidative stress [12,13,14]. Despite advancements in prevention strategies and effective antiviral treatments, HBV and HCV remain significant risk factors for HCC [15]. However, in recent years, the increasing prevalence of NAFLD and ALD has changed the landscape of HCC etiology [16]. This shift reflects changing lifestyle habits and the growing impact of environmental factors on public health. A study on the global burden of HCC in 2020 projected a concerning trend: without intervention, the number of new cases and deaths from HCC is expected to increase by over 55% by 2040 [17].

**Figure 1:**
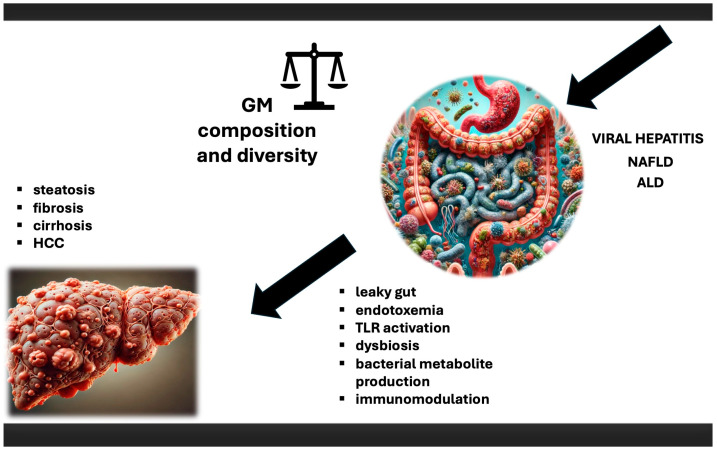
The role of microbiota in HCC. Impact of viral hepatitis, NAFLD, and ALD on microbiota, and subsequent consequences, which lead to chronic liver disease, with HCC development as a final stage. ALD—alcoholic liver disease; GM—gut microbiota; HCC—hepatocellular carcinoma; NAFLD—nonalcoholic fatty liver disease; TLR—Toll-like receptor.

Gut microbiota (GM) forms a complex and dynamic ecosystem, comprising viruses, fungi, protozoa, archaea, and predominantly bacteria, all existing in a finely tuned symbiosis with each other and the human host. It plays a critical role in human physiology, and disturbances (dysbiosis) in this delicate balance are widely recognised as contributors to various pathological conditions, including CLD, both infectious and non-infectious in nature. As a “virtual metabolic organ”, it establishes axes with several extra-intestinal organs, including the brain, lungs, cardiovascular system, kidneys, and especially the liver. The renowned gut-liver axis, resulting from the close anatomical and functional relationship between the gastrointestinal tract and liver, operates primarily through the portal circulation. This axis is further regulated by a complex network of interactions involving metabolic, immune, and neuroendocrine signalling [18]. A growing body of evidence from animal models and human studies suggests that the GM plays a pivotal role in the HCC development. Several mechanisms are thought to contribute to this process: (a) “leaky gut”, endotoxemia, and Toll-like receptor (TLR) activation, (b) dysbiosis and bacterial metabolite production, and (c) immunomodulation [18] (Figure 1). GM regulates the integrity of the intestinal barrier, the largest and very dynamic interface between the internal body and external environment. Disturbances in intestinal permeability, commonly known as “leaky gut”, allow microbes and their metabolites to enter the bloodstream, potentially triggering inflammation, leading to liver damage, and, consequently, carcinogenesis [19,20]. Bacteria and microbial antigens that colonize and interact with epithelial cells and host immunity exemplify the direct mechanisms of GM-induced carcinogenesis. In contrast, indirect mechanisms involve GM metabolites [21]; in fact, many metabolites, particularly short-chain fatty acids (SCFAs), bile acids (BAs), and intestinal hormones such as glucose-dependent insulinotropic peptide and glucagon-like peptide-1 (GLP-1), have been implicated as indirect agents in the development and progression of liver diseases. These metabolites are a relevant part of metabolic regulation and inflammation [22]. GLP-1 is a hormone that enhances insulin secretion, playing a crucial role in glucose metabolism and fat storage in the liver [23]. In contrast, GLP-1 also has anti-inflammatory properties that may protect against liver damage [24]. By regulating appetite and weight, these hormones impact metabolic health [25]. Additionally, SCFAs, especially butyrate, are well known for their ability to decrease liver inflammation. Overall, these metabolic compounds play significant roles in local and systemic immunomodulation by promoting the differentiation of regulatory T cells and enhancing antitumor immunity [26,27,28].

Growing interest in microbiota research has recently shifted focus toward the study of intratumoural microbiota, referred to as the “oncobiome” [29], which primarily originates from the local microbiome of the tumour-bearing tissue or translocates from distant sites, such as the gut [30]. This exploratory field has rapidly gained traction, offering new insights that allow us to delve deeper into the role microorganisms play in oncogenesis. Previously, the liver was widely believed to be a sterile organ, shielded from direct microbial influence. This revelation has opened many doors, though we are still only beginning to explore its full implications [31].

## 2. Viral Shadows: Microbes in the Wake of Hepatitis

HCV, an RNA virus from the *Flaviviridae* family, and HBV, a DNA virus belonging to the *Hepadnaviridae* family, are both non-cytopathic viruses with strong hepatic tropism, causing liver inflammation that can progress to CLD [32]. Chronic infections with HBV and HCV are the primary causes of HCC, accounting for approximately 60–70% of cases [33]. It is estimated that 55–58% of individuals infected with HCV develop chronic hepatitis C (CHC), with 20–30% of these cases progressing to liver failure or cirrhosis. Among patients with HCV-related cirrhosis, the annual HCC risk is approximately 1–4%. Notably, even in the absence of cirrhosis, 1–3% of HCV-infected individuals may still develop HCC over a 30-year span [34]. In comparison, about 95% of infants and 30% of children aged 1–5 years who are infected with HBV become chronically infected, while this rate drops to 5% in adults [35]. If left untreated, approximately 20% of these individuals are at risk of developing cirrhosis [36]. The annual HCC incidence in HBV patients without cirrhosis is estimated to be less than 1%, but this increases to 2–3% in those with cirrhosis [37].

GM dysbiosis is closely associated with chronic liver inflammation caused by these two viruses, as they trigger immune responses, release pro-inflammatory cytokines, and compromise gut barrier integrity and gut-liver communication networks [38]. In detail, in cirrhosis, impaired blood flow and liver dysfunction hinder detoxification processes, leading to bacterial overgrowth and systemic inflammation that affect interactions between immunity and GM [39]. Delayed intestinal transit fosters bacterial overgrowth, contributing to the development of small intestinal bacterial overgrowth (SIBO) and worsening dysbiosis. Both HBV and HCV also promote oxidative stress in liver cells, producing toxic byproducts that damage hepatocytes and affect gut epithelial cells [38]. Additionally, CHC can impact GM composition through IgA production by HCV-infected gastric B-lymphocytes [40]. Finally, the pivotal BAs role in maintaining GM homeostasis is disrupted due to their impaired production and secretion by the damaged liver [38].

On the other hand, an imbalanced GM and compromised intestinal barrier can lead to the translocation of gut microbes, potentially resulting in liver “invasion” [41]. Dysbiosis also has a significant impact on viral-host cell interactions, viral replication, modulation of TLR/Nod-like receptor activation, nuclear factor kappa B (NF-κB) signalling, the Janus kinase/signal transducer, activator of transcription (JAK/STAT) pathway, and CD4+T cell activation [34,40]. The release of pathogen-associated molecular patterns triggers inflammatory responses, with inflammation extending beyond the gut and impacting the liver as well, due to the close connection between the two organs via portal circulation [42]. The hepatic portal vein returns about 95% of BAs to the liver after they are reabsorbed in the terminal ileum. The GM deconjugates, dehydroxylates, and dehydrogenates the remaining 5% of BAs, forming secondary BAs (such as lithocholic acid (LCA) and deoxycholic acid (DCA)), which then enter the liver and move into the portal circulation [43]. This conversion is mediated by different gut bacteria, mainly *Clostridiales*. Kakiyama et al. documented that a reduction in primary to secondary BA conversion promotes more severe forms of liver disorders in HCV-related CLD [44]. Furthermore, it has been shown that age-specific seroclearance in chronic hepatitis B (CHB) is influenced not only by the maturation of the immune system but also by GM stability, a finding supported by animal studies [40]. Adult mice with a mature GM were able to clear HBV six weeks after infection, while adult mice whose GM had been sterilized with antibiotics remained HBV-positive. This highlights the GM’s role in anti-HBV immunity [45]. In previous reviews [40,46], we discussed distinct microbial profiles associated with HBV and HCV infections across various stages of viral-related CLD. In detail, CHB patients exhibit a higher abundance of the *Anaerostipes* genus compared to healthy controls. Cirrhosis linked to CHB is marked by reduced levels of *Bifidobacteria*, *Lactobacillus*, *F. prausnutzii*, and *E. faecalis*, alongside elevated levels of *Enterococcus*, *Enterobacteriaceae*, and *Streptococcaceae*. Moreover, in HBV-related HCC, there is a prominent decrease in *Proteobacteria*, *Prevotella*, *Faecalibacterium*, *Pseudobutyrivibrio*, *Lachnoclostridium*, and *Ruminoclostridium*, with an increase in *Escherichia-Shigella* and *Enterococcus*. In the context of HCV infection, the microbial composition in CHC patients shows an increased presence of *Enterobacteriacea*, *Prevotella*, *Coriobacteriaceae*, *Megasphaera*, *Succinivibrio*, and *Ruminococcaceae*, while *Bacteroides* and *Streptococcus* are reduced. CHC patients with fibrosis demonstrate a decreased abundance of *Akkermansia muciniphila*, while in CHC-related cirrhosis, there is a lower abundance of *Bacteroidetes* and higher levels of *Prevotella*, *Enterococcus*, *Staphylococcus*, *Veillonella*, *Proteobacteria*, *Megasphaera*, *Burkholderia*, and *Fusobacteria*. A high presence of *Escherichia coli* is associated with HCC. Additionally, Yun et al. reported that subjects in the CHB group with normal ALT (NALT) levels had a GM notably different from those in the high ALT level group, yet very similar to that of healthy volunteers [47]. In detail, they demonstrated that the *Megasphaera* genus, belonging to the phylum *Firmicutes*, was relatively more abundant in the high ALT group than in the NALT group. Research suggests that *Megasphaera* spp., known for their bile resistance and host-specific adaptation, are a significant component of the rumen microbiome and have positive effects on the host [48]. In contrast, the authors presented a different perspective, specifically noting that microbial diversity and the abundance of *Lactobacillus*, *Clostridium*, and *Bifidobacterium* were lower in CHB-NALT patients compared to healthy controls [49].

The belief in the sterility of tumours, especially solid ones, has lost credibility with the discovery of the oncobiome as a relevant TME component and its complex role in carcinogenesis and treatment sensitivity [50]. The microbiota colonising the liver may contribute to mutual TME regulation by influencing the recruitment of suppressive cells, such as myeloid-derived suppressor cells. Compared to gut bacteria, intratumoural microbes have a more direct and localised impact on tumour development. Using FISH technology, He Y et al. evaluated the HCC microenvironment and verified the bacterial presence within immune cells [51]. Based on a 16S rRNA sequencing approach, they evaluated 99 HCC and paracancerous tissues, producing a thorough map of the microbial communities present in these tissues. The results demonstrated that, compared to paracancerous tissues, the alpha and beta diversity of the microbial community were significantly higher in HCC tissues. In particular, they identified 11 bacterial genera with significantly different abundances between the tumoral and paracancerous groups, with 4 of these genera showing significantly elevated abundances in HCC. Additionally, they showed that the TME of HBV-related HCC tissues exhibited more diverse infiltrating bacterial colonies compared to non-HBV-related HCC tissues. Specifically, the HBV group had reduced abundances of *Dietzia* and *Oscillibacter*, while the abundances of *Veillonella* and *Alloprevotella* were increased. Furthermore, metabolic function predictions showed that the thiamin diphosphate biosynthesis pathway was blocked in the HBV group, while glutamate degradation and diacylglycerol production were greatly elevated. Finally, Komiyama et al. found that *Ruminococcus gnavus* (*R. gnavus*) was linked to the tumour area in patients with viral-related HCC. *R. gnavus* produces glucorhamnan, which binds to TLR4 and stimulates dendritic cells to produce TNF-α. Since TNF-α promotes HCC carcinogenesis, *R. gnavus* may represent a significant contributing factor in the viral-related HCC development [52].

## 3. The Silent Infiltratos: The Microbiota’s Role in NAFLD

NAFLD is characterized by an abnormal fat buildup in over 5% of hepatocytes. The term encompasses a wide range of conditions, including nonalcoholic fatty liver (NAFL), nonalcoholic steatohepatitis (NASH), and cirrhosis resulting from NAFLD. NASH represents the inflammatory form, characterised by fat accumulation, liver cell injury (ballooning), and inflammation, which may or may not be accompanied by fibrosis. NAFLD-related cirrhosis involves cirrhosis with either current or past signs of steatosis or steatohepatitis. Key risk factors for NAFLD include insulin resistance and metabolic syndrome, defined by the presence of three or more of the following: obesity, type 2 diabetes, high blood pressure, low levels of HDL cholesterol, and elevated triglycerides. Obesity is the most frequent risk factor, though NAFLD can also occur in individuals with normal or lower body weight, termed lean or non-obese NAFLD. In 2019, a group of 32 experts recommended the term metabolic (dysfunction)-associated fatty liver disease (MAFLD) to better reflect the diseases’ underlying causes [53]. The prevalence has been steadily rising, now affecting up to 30% of the global population. Combined with the recent success in treating HCV and the protective role of vaccination against HBV, NAFLD has become the fastest-growing cause of HCC. Notably, NAFLD-related HCC is five times more likely to develop during the pre-cirrhotic stage compared to other CLD causes. A significant issue is the lack of adequate screening for these patients, as is often detected outside the typical surveillance programs, more so than other liver disease etiologies [54].

NAFLD results from a combination of factors, including genetic predisposition, oxidative stress, immunity, and GM imbalances [55]. Fat accumulation in the liver is generally considered the starting “hit”. Over time, the continued lipid synthesis and uptake in the liver lead to increased oxidative stress and inflammation [56]. Recent studies have highlighted distinct microbiome characteristics associated with NAFLD, emphasising the role of dysbiosis in its progression to HCC [57,58]. Upon the disruption of intestinal barrier integrity and the leakage of microbes and their metabolites, an inflammatory cascade is activated, leading to the cytokines’ production, including IL-1β and IL-18, which contribute to liver damage [59,60]. This activation also sustains mitogenic signalling through the mitogen-activated protein kinase signalling cascade, with the extracellular signal-regulated kinase pathway playing a critical role [61]. Chronic exposure of TLRs to gut-derived metabolites maintains low-grade local inflammation, worsening NAFLD progression [62], which can lastly lead to HCC in some cases [63,64]. Moreover, the NAFL progression to NASH has been linked to GM dysbiosis and the loss of beneficial bacterial metabolic activities, such as SCFAs and vitamin production, roles in BA and amino acid metabolism, detoxification, and dietary fibres fermentation [65]. Overall, metabolic dysfunctions in NAFLD, such as insulin resistance and lipotoxicity, together with a “leaky gut”, increase the influx of microbial products to the liver, exacerbating inflammation. These factors further intensify dysbiosis, underscoring the peculiar interaction between GM and the progression of liver disease. Changes in genetic composition at various taxonomic levels have been documented, with a decrease in *Bacteroidetes* and an increase in *Firmicutes* and *Proteobacteria* [66,67,68]. At the family level, *Enterobacteriaceae* increase, while *Rikenellaceae* and *Ruminococcaceae* decrease [65,69]. Behary et al. investigated the GM in patients with NAFLD-related cirrhosis, comparing those with and without HCC. They documented that dysbiosis was prevalent in NAFLD-cirrhosis patients, with significant compositional and functional changes occurring upon HCC development. Bacterial extracts from NAFLD-HCC patients induced an immunosuppressive T cell phenotype, characterised by increased regulatory T cells and reduced CD8+ T cells, suggesting that the GM here exhibits a specific profile that can influence peripheral immune responses [70]. It is already established that *Helicobacter (H.) pylori* is associated with NAFLD [71,72,73,74]. Additionally, *Helicobacter hepaticus* has been identified as a key bacterium in promoting liver cancer in this patient group [75,76]. In the intestines, it could increase the risk without translocating to the liver to exert its effects. *H. hepaticus* can activate NF-κB-regulated networks associated with both innate and T helper 1 (Th1)-type adaptive immunity in the lower bowel and liver, indicating a systemic influence on immune responses [77]. Moreover, *Escherichia-Shigella* was found to be overabundant in patients with HCC compared with healthy controls. It produces pro-inflammatory lipopolysaccharides, which are known to contribute to hepatic fibrosis. In contrast, *Prevotella* was more abundant in the healthy controls, while *Bacteroides* species were more abundant in controls with NAFLD. The study highlighted that HCC-associated dysbiosis is more pronounced in patients with NAFLD than in patients with HBV, HCV, and ALD, suggesting that the GM may provide a potent noninvasive biomarker for early-stage HCC in this group [78]. Microbial signatures identified to date in NASH-derived HCC include *E. coli* [79], *Enterococcaceae, Lactobacillales, Bacilli*, and *Gammaproteobacteria* [80], as well as *Enterococcus, Limnobacter, and Phyllobacterium* [81], and a combination of 30 bacterial taxa serving as promising non-invasive biomarkers for early diagnosis [82]. Regarding GM metabolites, SCFAs influence lipid metabolism and glucose homeostasis, which are crucial in managing NAFLD [83]. BAs also play a significant role, acting as signalling molecules that activate various nuclear receptors in the liver (primarily farnesoid X receptor and G-protein-coupled bile acid receptor). This activation can impact both metabolism and cell proliferation [56,84]. However, abnormal BAs accumulation can lead to liver cell stress and apoptosis [85]. Hepatic translocation of obesity-induced lipoteichoic acid, a typical GM component of Gram+ bacteria, creates a tumour-promoting microenvironment through enhanced chronic inflammation, accelerated fibrosis progression, and increased tumour growth. LTA enhances the senescence-associated secretory phenotype (SASP) of HSCs. This process is further amplified by deoxycholic acid, another GM metabolite, which together upregulates the expression of SASP and cyclooxygenase-2 through TLR2 [86].

In addition to the oncobiome, Supply et al. presented evidence of bacterial DNA in healthy human liver. They found differences in the hepatic microbiome between obese and healthy lean individuals, proposing that alterations in the liver microbiome may serve as an additional risk factor for NAFLD development. Liver biopsies from the obese group showed lower alpha diversity at the phylum level, and the metagenomic profile showed a significantly higher abundance of *Proteobacteria* [87]. In addition, the bacterial DNA profile differed significantly between morbidly obese and non-morbidly obese patients. In morbidly obese patients, *Bacteroidetes* and *Firmicutes* were overrepresented, while *Proteobacteria*, specifically *Gammaproteobacteria*, *Alphaproteobacteria*, and *Deinococcus-Thermus*, were more abundant in the non-morbidly obese group [88,89,90]. Considering fibrosis stages, Champion et al. described the relevant role of liver bacteria in fibrosis progression, notably at the earliest stages. They discovered that 50% of liver taxa associated with early-stage fibrosis belonged to the *Enterobacteriaceae*, *Pseudomonadaceae*, *Xanthobacteriaceae*, and *Burkholderiaceae* families. *Flavobacteriaceae* and *Xanthobacteriaceae* were key in distinguishing between fibrosis stages F0 and F1 [91].

## 4. Alcohol’s Legacy: Microbiota Imprints in ALD

According to the Centers for Disease Control and Prevention (Atlanta, GA, USA) and the National Institute on Alcohol Abuse and Alcoholism (Bethesda, MD, USA), heavy drinking is defined differently for men and women. For women, it is consuming eight or more drinks per week or four or more drinks on any given day. For men, heavy drinking means 15 or more drinks per week or 5 or more drinks in a single day [92,93]. Ethanol is mainly absorbed in the intestines, with 90% metabolised in the liver, where it is converted into acetaldehyde and acetic acid. This metabolic process generates reactive oxygen species (ROS), which disrupt liver homeostasis. Most ethanol is oxidised by alcohol dehydrogenase (ADH) enzymes, while about 10–20% is metabolised through cytochrome P450 2E1 (CYP2E1). Acetaldehyde, a toxic byproduct, is further oxidised by aldehyde dehydrogenases into acetic acid, which is converted into acetyl-CoA for energy production and metabolism. Both ADH and CYP2E1 play crucial roles in ethanol metabolism. However, in heavy drinkers, CYP2E1 generates more acetaldehyde and ROS, contributing to liver damage. The excess nicotinamide adenosine dinucleotide produced during ethanol oxidation shifts metabolism toward lipogenesis, which can lead to fatty liver disease. Chronic alcohol consumption continuously produces ROS, exacerbating oxidative stress. These reactive molecules damage proteins and lipids, forming harmful byproducts, like malondialdehyde-acetaldehyde adducts, which may trigger immune responses. Moreover, increased CYP2E1 activity accelerates the metabolism of substances like acetaminophen, leading to toxic intermediates and increasing the risk of liver damage [94,95]. Long-term heavy drinking (over months and years) or binge drinking (more drinks in a short time) can damage nearly every organ, but the liver experiences the earliest and most severe tissue injury [94].

The ALD pathophysiology is multifactorial, involving ethanol-induced liver damage, immune system activation, disruption of intestinal barrier function, and GM alterations. In detail, acute ethanol consumption triggers oxidative, nitrative, and endoplasmic reticulum stress, inflammation, and cell death, while chronic consumption disrupts lipid metabolism, causing fat accumulation in the liver and altering the gut-liver axis by damaging the intestinal epithelium. Several factors influence ALD progression, including drinking patterns, beverage type, gender, ethnicity, metabolic syndrome, coexisting liver disorders, genetic factors, age, drugs, and smoking [93,94,95,96,97,98,99]. Alcoholic steatosis, the earliest indication of liver damage, is present in 95–100% of cases. Fortunately, it is completely reversible if alcohol consumption ceases. This condition develops through various mechanisms. Beyond changes in the redox potential and promoted lipogenesis, factors like adipose tissue also contribute to steatosis. Overall, lipids in hepatocytes are mainly stored in droplets, which are typically broken down through lipophagy—a process impaired by chronic alcohol consumption. This reduces the liver’s ability to oxidise fatty acids and export triglycerides and cholesterol through very low-density lipoproteins, leading to further fat accumulation in the liver. Approximately 10–35% of individuals who continue drinking develop lobular inflammation, which can progress to steatohepatitis. This stage may slowly advance to fibrosis in 20–40% of the cases and to cirrhosis in 8–20%. Initially, fibrosis is reversible, but ongoing alcohol consumption leads to persistent scarring. Finally, about 2% of patients with cirrhosis develop HCC [93,94,98,100].

Considering that only the aforementioned percentage of heavy drinkers develop severe forms of ALD and that the other triggers, aside from direct alcohol impact, are not fully understood, it is widely recognised that the GM may play a role. Supporting this, fecal microbiota transplantation (FMT) from patients with alcoholic hepatitis, when introduced to alcohol-fed germ-free mice, resulted in the migration of cytolisin-positive *Enterococcus faecalis* from the intestines to the liver, causing significant liver damage. In contrast, mice that received FMT from patients without alcohol hepatitis exhibited much less liver damage [101]. GM dysbiosis can both exacerbate liver injury and impair the body’s ability to repair liver damage [92,102,103]. Spatz et al. conducted an interesting study on the role of the BA receptor TGR5 (or GPBAR1) and GM involvement in alcohol-induced liver injury in mice. They demonstrated that TGR5 deficiency exacerbates alcohol-induced liver lesions by altering GM composition, while alcohol further aggravates GM dysbiosis. These findings underscore the GM role in alcohol-induced liver inflammation and steatosis. In addition, FMT experiments confirmed that the worsening of liver damage in dysbiotic mice is at least partially linked to GM changes [104]. More recent findings by Liu et al. emphasise the role of apolipoprotein H (APOH) deficiency in disrupting BA metabolism in ALD through GM alterations. APOH deficiency, especially in alcohol-fed mice, significantly altered BA composition and increased liver damage. This was marked by increased levels of harmful BAs, like TCA and DCA, contributing to liver steatosis [105]. Strategies to restore “healthy microbiota” and gut-liver homeostasis have been explored, including probiotics, prebiotics, FMT, and antibiotics. Probiotics and prebiotics may help restore beneficial microbial populations, reduce gut permeability, and attenuate liver inflammation. In early-stage research, patients with alcoholic hepatitis who received FMT showed higher survival rates, offering a promising therapeutic option [106,107].

There is a general decline in bacterial diversity within the microbiome of patients suffering from all forms of ALD. When focusing on specific bacterial groups, certain consistent patterns have emerged. Even in the absence of significant liver damage (early stages of ALD), alcohol consumption alone is linked to a reduction in *Bacteroidaceae* and a broader increase in *Proteobacteria*. Alcohol-related cirrhosis is associated with a marked decrease in *Lachnospiraceae* and *Ruminococcaceae*, families known for producing SCFAs and being regarded as beneficial to gut health. Additionally, a consistent decline in *Clostridiales XIV* and *Blautia* has been observed. Pathogenic bacteria, such as *Enterobacteriaceae* and *Streptococcaceae*, are elevated in alcoholic-related cirrhosis, as is the genus *Enterococcus*. Moreover, an increase in the typically beneficial genera *Bifidobacterium* and *Lactobacillus* has been documented, while the rise in *Veillonella* appears less consistent. In addition, groups with alcohol-related conditions show a significant decrease in fungal diversity, alongside a pronounced increase in *Candida species* and a reduction in *Epicoccum*, unclassified fungi, *Galactomyces*, and *Debaryomyces* [108,109,110]. Zhong et al. conducted a study examining the GM structure and composition in both early and advanced stages of ALD (steatosis vs. cirrhosis) patients. Their research confirmed notable shifts in microbial diversity and composition as the disease progressed, with *Streptococcus* identified as the dominant bacterium in ALD patients. Significant changes in both alpha and beta diversity were observed, even in the early stages of the disease. Furthermore, the incidence of SIBO was found to be nearly three times higher in ALD patients compared to non-alcoholic controls [111]. Bajaj et al. highlighted that the shift in the balance between bacteria in patients with liver cirrhosis is captured by the cirrhosis dysbiosis ratio. This ratio is calculated by dividing the combined abundance of *Lachnospiraceae* and *Ruminococcaceae* by the abundance of the *Enterobacteriaceae* and *Bacteroidaceae*. A lower ratio indicates more severe GM disruption in patients with alcoholic liver cirrhosis [112]. A study by Seok et al. provides valuable insights into the microbial composition and diversity in three groups of patients: (1) alcoholic liver cirrhosis (ALC), (2) alcoholic HCC (A-HCC), and (3) healthy controls (HCs). Notably, *Proteobacteria* were markedly increased in both ALC and A-HCC groups compared to the HC. In contrast, *Bacteroidetes* were decreased in the same groups, while *Firmicutes* showed no significant changes. Regarding alpha and beta diversity, ALC and A-HCC groups exhibited similar microbial diversity profiles, but there was a statistically significant difference between the HC and disease groups. At the genus level, the study revealed an enrichment of *Lactobacillus* in both the ALC and A-HCC groups, with a significant increase in abundance compared to the HC. Interestingly, genera such as *Alistipes*, *Butyricimonas*, *Mucispirillum*, *Oscillibacter*, *Parabacteroides*, *Paraprevotella*, and *Prevotella* were enriched across the diseased groups, suggesting potential microbial signatures associated with these pathologies. Moreover, species like *Akkermansia muciniphila*, *Bacteroides fragilis*, *Parabacteroides distasonis*, and *Alistipes shahii* were significantly enriched, reinforcing the notion of distinct microbial alterations linked to alcoholic liver disease [113]. Studies on liver tissue samples across various etiologies remain limited, and this is also the case for alcohol-related HCC, which could deepen our understanding of the oncobiome in this patient group. However, there is no doubt that continued research will provide valuable insights, even as it broadens the scope of inquiry and raises new questions.

## 5. Conclusions: Navigating the Microbes in Liver Disease

There is a dynamic interaction between the microbial ecosystem and liver tissue, particularly in CLD. The identification of specific dysbiosis patterns in viral hepatitis, NAFLD, and ALD (Table 1 and Table 2), associated with various stages of liver disease, provides insights into the mechanisms driving liver inflammation, immune modulation, and fibrosis. These microbiota-driven mechanisms have emerged as critical factors in the HCC development. Once thought to be sterile, the discovery of intratumoural microbiota within HCC tissue adds another layer of complexity. The emerging concept of an “oncobiome” within liver tumours, along with the growing understanding of the gut-liver axis, offers a promising avenue for future innovations. The adage “prevention is better than cure” should guide all pathologies, including liver carcinoma, emphasising the relevance of focusing research efforts on the earlier stages of disorders that lead to HCC. As personalised medicine becomes more prevalent, the microbiome could serve as an additional diagnostic biomarker for identifying patients at risk of advanced liver disease, enabling early intervention and improved clinical outcomes. Due to its non-invasiveness, high efficiency, and accuracy, the GM holds significant potential as a diagnostic biomarker, improving disease management and outcomes. Overall, microbial shifts in liver disease are not just markers but active contributors to disease progression. The therapeutic potential of modulating the microbiota-whether through probiotics, prebiotics, FMT, or microbiome-based drugs-seems a promising approach to reducing liver damage or at least preventing progression to more severe stages. This is especially relevant during steatosis and early fibrosis, which are still reversible conditions.

## Figures and Tables

**Table 1:** Etiology-dependent gut microbiota composition in different CLD stages.

GM	Liver Steatosis/Fibrosis	Liver Cirrhosis	HCC
HBV/HCV	**Family:** ↑ Coriobacteriaceae ↑ Ruminococcaceae ↑ Enterobacteriaceae **Genus:** ↑ *Anaerostipes* ↑ *Prevotella* ↑ *Megasphaera* ↑ *Succinivibrio* ↓ *Bacteroidetes* ↓ *Streptococcus* **Species:** ↓ *Akkermansia muciniphila*	**Phylum:** ↓ Bacteroidetes ↑ Proteobacteria ↑ Fusobacteria **Family:** ↑ Enterobacteriaceae ↑ Streptococcaceae **Genus:** ↓ *Bifidobacteria* ↓ *Lactobacillus* ↑ *Enterococcus* ↑ *Prevotella* ↑ *Staphylococcus* ↑ *Veillonella* ↑ *Megasphaera* ↑ *Burkholderia* **Species:** ↓ *F. prausnutzii* ↓ *E. faecalis*	**Phylum:** ↓ Proteobacteria **Genus:** ↑ *Veillonella* ↑ *Escherichia-shigella* ↑ *Enterococcus* ↓ *Prevotella* ↓ *Faecalibacterium* ↓ *Pseudobutyrivibrio* ↓ *Lachnoclostridium* ↓ *Ruminoclostridium* **Species:** ↑ *Ruminococcus gnavus*
NAFLD	**Phylum:** ↑ Proteobacteria ↑ Firmicutes **Family:** ↓ Bacteroidaceae	**Family:** ↑ Xanthobacteriaceae ↑ Flavobacteriaceae	**Phylum:** ↑ Proteobacteria **Family:** ↑ Enterobacteriaceae ↓ Prevotellaceae ↑ Streptococcaceae
ALD	**Phylum:** ↑ Proteobacteria **Family:** ↓ Bacteroidaceae	**Phylum:** ↓ Bacteroidetes ↑ Proteobacteria **Family:** ↓ Lachnospiraceae ↓ Ruminococcaceae ↑ Enterobacteriaceae ↑ Streptococcaceae **Order:** ↓ Clostridiales XIV **Genus:** ↓ *Blautia* ↑ *Enterococcus* ↑ *Bifidobacterium* ↑ *Lactobacillus* ↑ *Candida* spp. ↓ *Epicoccum* ↓ *unclassified fungi* ↓ *Galactomyces* ↓ *Debaryomyces*	**Phylum:** ↑ Proteobacteria ↓ Bacteroidetes **Genus:** ↑ *Lactobacillus* ↑ *Alistipes* ↑ *Butyricimonas* ↑ *Mucispirillum* ↑ *Oscillibacter* ↑ *Parabacteroide* ↑ *Paraprevotella* ↑ *Prevotella* ↑ *Candida* spp. ↓ *Epicoccum* ↓ *unclassified fungi* ↓ *Galactomyces* ↓ Debaryomyces **Species:** ↑ *Akkermansia municiphila* ↑ *Bacteroides fragilis* ↑ *Parabacteroides distasonis* ↑ *Alistipes shahii*

GM—gut microbiota, HCC—hepatocellular carcinoma, NAFLD—non-alcoholic fatty liver disease, ALD—alcoholic liver disease. ↑ indicates an increase, while ↓ indicates a decrease in abundances.

**Table 2:** Etiology-dependent oncobiome composition in hepatocellular carcinoma.

Oncobiome	Phylum	Family	Genus
HBV/HCV	↑ Bacillota ↑ Firmicutes ↓ Actinomycetota	↑ Oscillospiraceae ↑ Acidaminococcaceae ↓ Dietziaceae	↑ *Veillonella* ↑ *Alloprevotella* ↓ *Dietizia* ↑ *Oscillobacter* ↑ *Ruminococcus*
NAFLD	↑ Proteobacteria ↑ Bacteroidetes ↑ Deinococcus Thermus ↑ Firmicutes ↑ Pseudomonadota	↑ Pseudomonadaceae ↑ Xanthobacteriaceae ↑ Burkholderiaceae ↑ Enterobacteriaceae	

NAFLD—non-alcoholic fatty liver disease. ↑ indicates an increase, while ↓ indicates a decrease in abundances.

## Data Availability

No new data were created or analysed.
